# Novel Interdisciplinary Enhanced Recovery after Surgery Protocol Implementation in Paediatric Orthopaedics

**DOI:** 10.3390/jpm13091417

**Published:** 2023-09-21

**Authors:** Sławomir Zacha, Aleksander Szwed, Jakub Miegoń, Karolina Skonieczna-Żydecka, Agata Andrzejewska, Elżbieta Modrzejewska, Marcin Horecki, Konrad Jarosz, Jowita Biernawska

**Affiliations:** 1 Department of Paediatric Orthopaedics and Musculoskeletal Oncology, SPSK nr 1 Pomeranian Medical University, 71-252 Szczecin, Poland; 2 Department of Anaesthesiology and Intensive Therapy, SPSK No 1 Pomeranian Medical University, 71-252 Szczecin, Poland; jakub.miegon@gmail.com (J.M.); lisienko@wp.pl (J.B.); 3 Independent Biochemical Sciences, Pomeranian Medical University, 71-460 Szczecin, Poland; 4 Department of Infectious Diseases, Tropical Diseases and Acquired Immunodeficiencies, Pomeranian Medical University, 70-204 Szczecin, Poland; 5 Department of Clinical Nursing, Pomeranian Medical University, 70-204 Szczecin, Poland

**Keywords:** enhanced recovery after surgery (ERAS), paediatric orthopaedics, cryoanalgesia, perioperative period

## Abstract

The enhanced recovery after surgery (ERAS) protocol is not routine management in paediatric orthopaedics. Cultural differences or assumptions about the financing of medical care in different countries encourage creative adaptation of general assumptions in local communities. The aim of this study was to compare the quality of the perioperative period before and after the introduction of an interdisciplinary protocol adopted to organisational conditions. A group of 4098 children were included in the “before–after” observational study. The data of 1553 patients (BEFORE group) were analysed in terms of compliance with the enhanced recovery after surgery protocol guidelines and the time and cost of hospitalisation over a 20-month period. A novel interdisciplinary protocol was developed, including an education and training app called BackOnFeet (BOF^®^), standardised hospital management, and the introduction of methods novel to Poland (intraoperative nerve cryoanalgesia in children). A further 2545 patients (AFTER group) were reassessed over a period of 20 months. It was found that the groups differed in hospitalisation time (*p* < 0.001), type of procedures, and percentage ratio of costs incurred to revenue generated. The usefulness of the BOF^®^ app as an effective educational tool was demonstrated. The optimisation of perioperative management in paediatric orthopaedics based on novel tools and the interdisciplinary ERAS protocol is possible and brings tangible benefits in psychological, organisational, and financial terms.

## 1. Introduction

Surgical intervention causes disruption to homeostasis and generates stress on the body. Depending on the initial general state of the patient and the perioperative care, the patient’s recovery time in hospital may be unnecessarily prolonged. The integrated protocol has a real impact on reducing the risk of ‘avoidable’ complications and thus the length of hospitalisation. The idea of a comprehensive perioperative care protocol for enhanced recovery after surgery (ERAS) is to optimise the patient’s condition in order to shorten the recovery period (http://www.erassociety.org) (accessed on 24 August 2023) [1]. The use of the ERAS protocol has well-documented effects in general surgery, as well as in some orthopaedic procedures (hip and knee alloplasty, lumbar spinal fusion) [2,3,4]. It has proven benefits in the adult patient population, mainly due to the advancement of comorbidities [5,6,7]. There are few studies in the paediatric orthopaedic population [8,9]. They mainly concern the use of the ERAS protocol during scoliosis correction [10,11,12,13,14,15].

The ERAS protocol is not routinely used in paediatric orthopaedics. Therefore, in the Department of Paediatric Orthopaedics and Oncology of the Musculoskeletal System of the Pomeranian Medical University, Clinical Hospital No 1 in Szczecin, Poland, a project was created to adapt the ERAS guidelines to the specifics of paediatric orthopaedics under Polish conditions. There is no common protocol of management regarding paediatric orthopaedic procedures in Poland. The project enabled each point of the ERAS protocol to be adapted to current guidelines applicable to perioperative care in children and to be used in daily clinical practice.

The aim of this study is to compare the course of the perioperative period based on an analysis of the medical records of procedures performed in previous years to the treatment results obtained after the creation and implementation of a multidisciplinary protocol consistent with the ERAS protocol and the guidelines in paediatrics.

## 2. Materials and Methods

This study was conducted in accordance with the Declaration of Helsinki, and the protocols for novel procedures were approved by the Institutional Review Board at Pomeranian Medical University in Szczecin (resolution no. KB 0012/126/10/2021/Z and KB-006/43/2022).

A group of 4098 children undergoing surgery in the Department of Paediatric Orthopaedics of the Pomeranian Medical University, Poland, was included in the “before–after” observation study.

The ‘BEFORE’ group included a further 1553 patients undergoing paediatric orthopaedic surgery operated on within 20 months of 2018. In this group, there were no standardised guidelines for surgical technique, anaesthesia, postoperative pain management or rehabilitation plan, and the therapeutic process was based on the decision of the attending specialist.

After analysing the previous practice in terms of compliance with the ERAS protocol guidelines and the time and cost of hospitalisation, we created an innovative interdisciplinary protocol for the management of patients undergoing surgery. The project included the following aspects:

- Qualification and preparation for surgery and anaesthesia in the Outpatient Clinic,
- Pre-operative preparation of the patient: education via access to the proprietary educational and training app BackOnFeet, prehabilitation (physical activity, diet), and psychological consultation,
- Standardised hospital management of surgical techniques, modes of anaesthesia, options for advanced monitoring, multimodal analgesia and a plan for pain assessment and relief, including novel methods (intraoperative nerve cryoanalgesia),
- Creation of an established interdisciplinary team (orthopaedic surgeons, anaesthetists, physiotherapists, nurses, psychologists, and dieticians) with regular training to implement optimal perioperative care.

The principles of the project were introduced into everyday practice with the tools created. For patients and their families, a team of doctors and physiotherapists created a free mobile educational and training application called BackOnFeet (BOF^®^, Version 1.1, created by Sławomir Zacha and Algomedica Sp. z o.o., 71-422 Szczecin, Poland) with information on musculoskeletal diseases treated with surgery, methods of anaesthesia and pain relief, prehabilitation and post-operative rehabilitation, together with videos dedicated to specific types of diseases and surgery. The patient and carers received reliable information and started learning exercises that were then used during early post-operative rehabilitation. The app is free of charge and available in Polish and English for download on the Apple App Store and Google Play platforms. The use of BOF^®^’s proprietary app for patients was analysed based on the results of a user survey (evaluation of usefulness, clarity of content, and degree of satisfaction). When we introduced the app in the “after group” we orally asked every patient about usefulness, clarity of content, and degree of satisfaction and we wrote down the answers on the form attached to the medical records of the disease history. 

Following orthopaedic qualification for surgery, patients and carers were presented with a detailed surgical treatment plan and given access to the BOF^®^ app. Patients were then consulted and qualified for anaesthesia by an anaesthetist in the outpatient clinic, where, in addition to the standard preparation depending on the patient’s condition, the type and extent of anaesthesia, pain assessment and relief plan, preoperative fasting, the possibility of consuming a carbohydrate-rich beverage, anxiety relief and anti-emetic management were determined.

In the hospital, patients were cared for by a psychologist and a dietician. After surgery, acute pain intensity at rest and during activity was regularly assessed and analgesics were administered at fixed intervals to reduce the risk of breakthrough pain and to allow early implementation of postoperative rehabilitation according to exercises learned before surgery.

The medical team was trained to introduce less invasive surgical techniques, especially in the treatment of scoliosis and congenital thoracic deformities. The surgical team established in advance a personalised surgical plan and the possibility of performing the procedure using a minimally invasive technique. Intraoperative neuromonitoring was routinely introduced during scoliosis correction surgery, as well as the selection of appropriate implants adapted to the morphology of the curvature and the age of the patient. Extensive corrective and reconstructive procedures were performed by two experienced surgeons and with the simultaneous use of two independent electrocoagulations. Procedures with a higher risk of complications were routinely accompanied by advanced haemodynamic monitoring techniques and a goal-directed therapy (GDT) protocol. Multimodal analgesia with ultrasound-guided regional anaesthesia was used whenever possible. Intraoperative intercostal nerve cryoanalgesia was performed for the first time in children in Poland, in May 2022, and it was added to specific types of surgery to reduce postoperative pain and prevent chronic pain [16].

After all the above measures were implemented, the results were reassessed in terms of perioperative compliance with ERAS guidelines and hospitalisation time and costs. For the analysis, a parameter comparing the costs incurred to the revenue generated was used.

The ‘AFTER’ group consisted of a further 2545 patients undergoing paediatric orthopaedic surgery over a 20-month period from September 2021.

Patient groups were analysed according to age groups (<1, 1–6, 7–18 years of age), gender, type of procedures performed, length of hospitalisation, revenue generated, and costs incurred. From both groups, the most frequently performed surgical procedure categories were additionally extracted:

Category 1: reconstructions of the osteoarticular system (osteotomies and long bone lengthening procedures, reconstructions of congenital and acquired bone and joint defects);
Category 2: multi-tissue reconstructions of osteoarticular and soft tissue defects (pelvic osteotomies with hip reconstruction, multi-tissue reconstructions of peripheral joints);
Category 3: spinal deformities (scoliosis of various aetiologies);
Category 4: other procedures.

The total income from the sale of procedures performed and the costs of obtaining it were analysed economically. For the analysis, a parameter comparing the costs incurred to the revenue generated was used. The average cost of a patient’s hospitalisation and the average revenue from billed procedures per patient were then calculated. The percentage of the average cost to the obtained revenue was also calculated.

**Figure 1:**
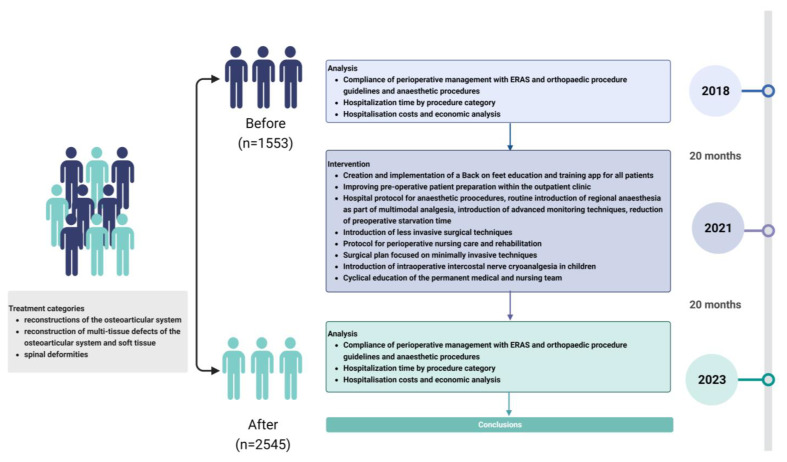
Study plan.

The plan for the observational study is shown in Figure 1.

Table 1 summarises the general ERAS protocol guidelines and proprietary tools for adaptation to the specificities of paediatric orthopaedic surgery for the entire perioperative period.

We performed statistical analyses with the use of Med Calc statistical software version 22.002 (Ostend, Belgium). Two-sided *p* < 0.05 indicated a statistically significant difference. Continuous data were expressed as mean ± standard deviation (SD). As for the number of data, we made assumptions according to the central theorem and performed analyses using the parametric test, i.e., *t*-test or Anova, as appropriate. To find the interaction between tested independent variables, two-way Anova was used. Categorical data were expressed as numbers (percentages) and compared using chi-square or Fisher’s exact test. Bonferroni correction was applied.

## 3. Results

The groups before and after the introduced intervention did not differ significantly in terms of gender. However, statistically significant differences were shown for the other parameters analysed. It was noted that in the “AFTER” group, children 7–18 years old were operated on more frequently. The results are shown in Table 2.

**Figure 2:**
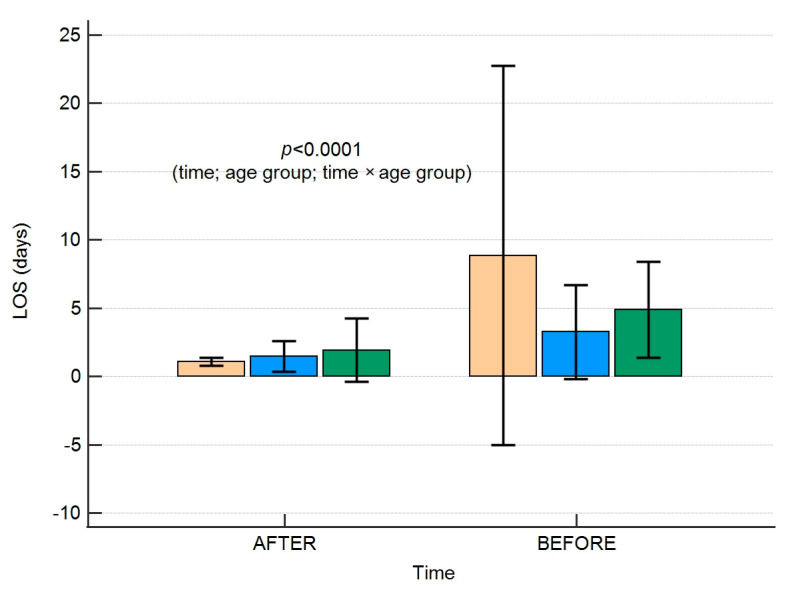
A box plot presenting the length of hospitalisation according to age groups in the time intervals studied. Colour of boxes corresponds to the age groups: orange <1 year, blue 1-6 years, and green 7-17 years. Error bars present standard deviations. LOS-length of hospital stay.

As evidenced by two-way Anova, the length of hospitalisation analysed in each age group was significantly shorter, irrespective of which age range was analysed (*p* < 0.001). We found a significant interaction between the time and age group. The results are shown in Figure 2.

**Figure 3:**
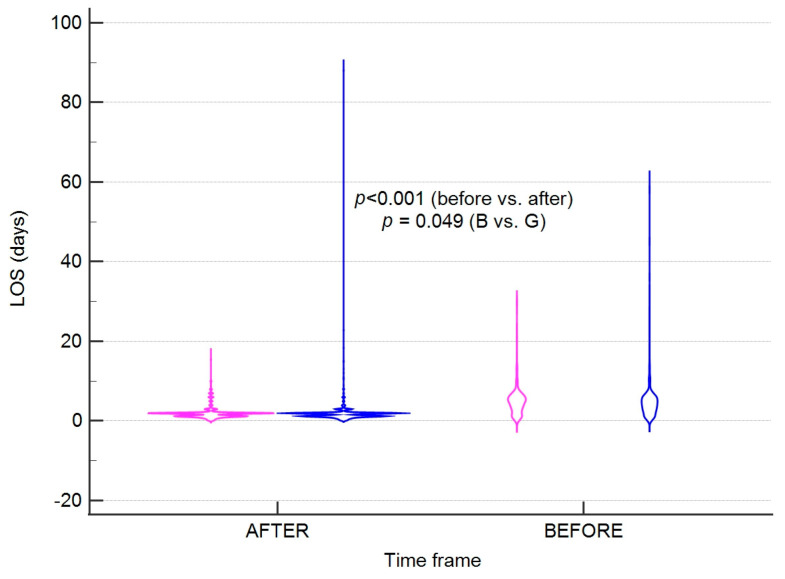
A violin plot presenting the length of hospitalisation according to gender in the time intervals studied. Colours correspond to gender; pink (G)-girls; blue (B)-boys. Error bars present standard deviations. LOS-length of hospital stay.

We did not find that the gender of the patients significantly affected the LOS in analysed time frames as shown in Figure 3.

**Figure 4:**
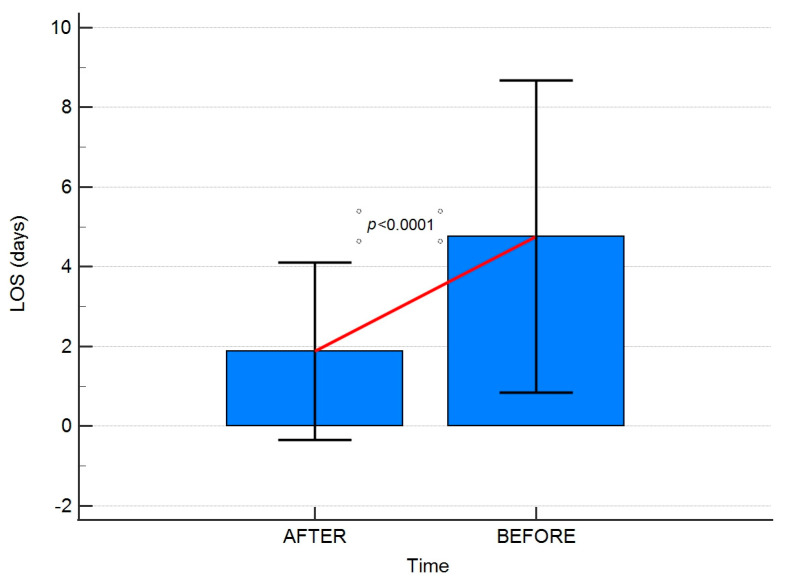
A box plot presenting the comparison of hospitalisation times in BEFORE and AFTER groups. Error bars present standard deviations. LOS-length of hospital stay.

**Figure 5:**
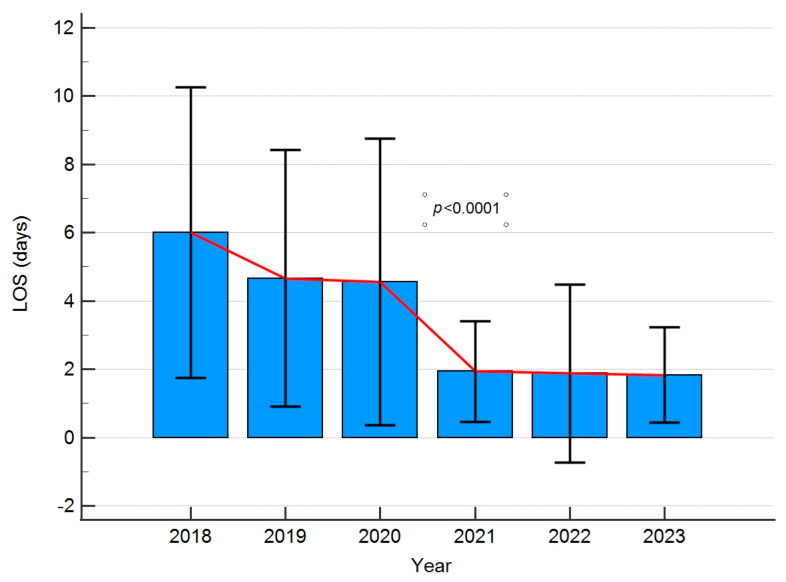
A box plot presenting the comparison of hospitalization times in the years studied. Error bars present standard deviations. LOS-length of hospital stay.

Significant differences in hospitalisation times were observed in the years following implementation of the guidelines. It is presented in Figure 4 and Figure 5.

In the BEFORE group, the assessment of compliance with ERAS protocol guidelines and standards showed the following:

- Lack of prehabilitation;
- No anaesthetic consultation in advance in the Outpatient Clinic area;
- Significantly prolonged preoperative starvation time and excessive fluid restriction (inability to consume carbohydrate-rich beverages before surgery);
- No pre-emptive analgesia;
- Incomplete use of multimodal analgesia;
- Non-routine use of regional analgesia;
- Lack of use of advanced haemodynamic monitoring techniques;
- Long-term use of opioids;
- Delayed start of post-operative rehabilitation due to pain;
- Delayed return to oral feeding.

All the above-mentioned points of the ERAS protocol were implemented and documented in the hospital electronic database in the AFTER group according to the established interdisciplinary perioperative protocol, taking into account modifications of the management in preparation for specific groups of procedures. Supervisors checked the compliance of the practice and documentation with the protocol. It was a team effort to achieve compliance. The use of our patient education and training app BOF^®^ was analysed based on the results of a user survey. There was widespread use of the BOF^®^ app by patients and carers based on the results of a survey included in the app. Users gave the highest rating (5/5 points) regarding the usefulness and accessibility of the content presented.

New surgical techniques introduced in the AFTER group included correction of congenital anterior chest wall deformities, correction of paediatric scoliosis using magnetic growing rods, vertebral body tethering (VBT), and lengthening of long bones using intramedullary growing nails. Intraoperative neuromonitoring was routinely introduced during scoliosis correction. The selection of appropriate surgical systems is adapted to the morphology of the curvature and the age of the patient. Extensive corrective and reconstructive procedures were performed by two experienced operators with the simultaneous use of two independent electrocoagulations to reduce operative time and ensure better haemostasis.

The entire team involved in perioperative care was trained in how to assess and relieve acute pain in the perioperative period via a series of mandatory training courses (www.leczbol.pl) (accessed on 24 August 2023).

Given the use of new surgical procedures, we implemented advanced anaesthesia and monitoring techniques. We introduced selective lung ventilation during thoracoscopic procedures for the treatment of anterior chest wall deformities and VBT scoliosis surgery. We implemented minimally invasive haemodynamic monitoring and goal-directed therapy (GDT) during scoliosis surgery, resulting in fewer neurological and cardiovascular complications and a reduction in hospitalisation time from 10 to 7 days within the AFTER group (*p* < 0.001). We introduced intercostal nerve cryoanalgesia procedures during Nuss surgery in patients with funnel-shaped anterior chest wall deformities. Hospitalisation time as a result of protocolised care and the use of intraoperative cryoanalgesia within the AFTER group was further reduced from six to four days (*p* < 0.011). A higher frequency of rehospitalisations was observed since the introduction of the new surgical procedures.

The percentage parameter comparing the costs incurred to the revenue received is shown in Table 3.

The percentage of average cost to revenue in the BEFORE group was 82.70%; in the AFTER group, it fell to 72.16%, while an increase of 78% in the average cost of treatment was observed, with a 104% increase in revenue.

## 4. Discussion

The idea of the ERAS protocol is to reduce patient stress, prevent the occurrence of postoperative complications, and reduce hospitalisation time and costs [2,3]. The highest efficacy of the protocol is best documented in the elderly population with multiple advanced comorbidities, undergoing advanced surgical procedures [3,5]. It is therefore difficult to demonstrate a measurable financial effect in a group of paediatric patients undergoing ‘minor’ paediatric orthopaedic procedures. In contrast, the use of the ERAS protocol for “major” procedures (e.g., correction of scoliosis) was proven in the literature [11,12,13,14,15]. The question arises whether it is worth applying the ERAS protocol to all paediatric orthopaedic surgical procedures.

The benefits of the ERAS protocol include broad psychological and organisational aspects [21,22,23,24,25]. Cultural differences or assumptions about the financing of medical care in different countries encourage creative adaptation of general assumptions in local communities. Nevertheless, Poland lacks such in the field of paediatric orthopaedics. The results of this publication provide an answer to this problem. By creating an interdisciplinary protocol and proprietary tools adapted to Polish organisational conditions, we succeeded in improving treatment outcomes. The result was a significant reduction in hospitalisation time, the introduction of innovative treatments and procedures, a reduction in costs and, above all, improved care, and satisfaction for patients, their families, and the entire treatment team. The results of this study show that the use of simple educational tools, cyclical training and feedback provide a sense of influence on the outcome of treatment. They thus increase the motivation to make a concerted effort aimed at the best possible outcome. The tools developed by the authors and the protocol are adaptable and can be used in other facilities.

### 4.1. Pre-Operative Period

Once the indication for surgery is established, the waiting time should be used for patient education, prehabilitation, and optimisation of the general condition. Under Polish conditions of healthcare organisation, this time is not used properly. There is no possibility for the patient to be educated by an interdisciplinary team (orthopaedic surgeon, anaesthetist, rehabilitation specialist, dietician, nurse, psychologist) on preoperative preparation for a sufficiently long time before surgery. Given the limitations of direct patient–doctor contact time and the impossibility of presenting all aspects related to the disease and hospitalisation, the solution may be the proprietary BOF^®^ application developed by our team. Its educational value and usefulness in reducing stress due to lack of knowledge are confirmed by all users. If the patient/family declared using the application, our team believed them. Now, we are working on modifying the app so that the user can complete the survey in the app and the answer will be sent to our protected database.

The benefits of learning disease-specific exercises in the home environment and then using them immediately after surgery are obvious. In addition, providing the patient with a reliable source of knowledge increases the sense of participation and further motivates effort by enabling the healing process to begin even before the operation. The authors of a systematic review confirm that the use of mobile applications has a positive impact on health-promoting behaviour and users’ sense of satisfaction, and it enables proper preparation for surgical procedures compared to conventional patient education [25,26,27].

The ability to assess the patient’s general condition, underlying disease and the severity of comorbidities, risk of anaemia, nutritional status, addictions, and types of medication taken in an outpatient setting in good time before the scheduled operation is crucial with regard to reducing the risk of complications. This reduces the number of hospitalisations during which surgery cannot be performed because the patient is not optimized [28]. This has a significant impact on unnecessary costs incurred. Changing the organisation of the anaesthetic consultation from a premedication evening visit on the eve of surgery to an outpatient anaesthetic clinic on a conveniently scheduled basis solves this problem.

The problem of prolonged preoperative fasting is common in Polish hospitals. The ERAS protocol recommends assessment of nutritional status, a standard-compliant starvation time and loading with clear carbohydrates 1–2 h before surgery. Failure to follow the ERAS recommendations increases the risk of a catabolic state, insulin resistance, an increased stress response to surgery, and thirst [25]. The education of staff in this area and the introduction of a carbohydrate intake rule before surgery have effectively addressed this problem.

### 4.2. Surgery-Anaesthetic Team

ERAS guidelines covering prophylactic antibiotic therapy prior to skin incision, prophylaxis of thromboembolic complications and prophylaxis of nausea and vomiting to reduce the risk of postoperative complications are supported by the dedicated guidelines [18]. The same is true of the recommendations for the assessment and management of pain in terms of multimodal analgesia and maintenance of normothermia and normovolemia [19]. Given the existence of these recommendations, their implementation is the responsibility of all members of the interdisciplinary team.

The results of the presented study indicate that, prior to the implementation of protocolised care, many of the recommendations from relevant scientific societies were not routinely applied. The phenomenon of non-compliance to clinical guidelines is widely reported in the literature [29,30]. The state of knowledge, experience, personal beliefs, and skills play a fundamental role. According to the authors, the current state of the actual use of recommendations can be improved by the active implementation of guidelines during regular team training and by increasing team motivation.

According to the literature, up to 70% of children experience acute pain in the perioperative period [19]. The causes are complex, such as a lack of pre-emptive analgesia and a lack of routine use of multimodal analgesia with regional analgesia [19]. The problem is not the availability of equipment, as the ultrasound machine is a permanent fixture in the operating theatre. The reason is the human factor. For this reason, the activities of the entire therapeutic team must be directed towards this aspect.

The reorganisation of the structure of the paediatric orthopaedics department presented in this project and the training of staff have enabled the implementation of advanced surgical and anaesthetic procedures. Advanced procedures in stressed patients are challenging for the anaesthetic team and inspire continuous training. Therefore, advanced techniques, including haemodynamic monitoring, are implemented in response to the implementation of extensive reconstructive, oncological, and spinal deformity surgeries, e.g., the optimisation of the patient’s condition based on minimally invasive haemodynamic monitoring and goal-directed therapy during these procedures enabled a reduction in neurological and cardiac complications and hospitalisation times. The slight increase in the cost of the procedure because of the use of additional equipment ultimately allows for a reduction in the total cost of treatment, as confirmed by both the study presented here and the results of numerous publications [20].

### 4.3. Surgery—Orthopaedic Team

During surgery, care should be focused on selecting minimally invasive techniques and reducing the anaesthetic time and the surgery itself to the minimum necessary. The surgical team should establish in advance a personalised plan for the surgical technique and the possibility of performing the procedure using the least invasive technique possible. In response to these considerations, the authors of this project have introduced changes to improve the safety of the procedure.

As a result of periodic training of all members of our therapeutic team in pain management, effective pain relief is no longer a challenge for anaesthetists alone. The entire team has become open to exploring alternative and innovative methods. An example is the implementation of cryoanalgesia of intercostal nerves during thoracic deformity correction in relation to standard regional analgesia. The proven benefits of intercostal nerve cryoanalgesia include a reduction in the severity of postoperative pain, the risk of persistent pain, and a reduction in the need for postoperative analgesics [16,31]. This translates into the possibility of a faster return to motor independence during rehabilitation, patient satisfaction, shorter hospitalisation time, and a reduction in overall treatment costs. We were the first in Poland to implement this procedure in children and confirm all the beneficial effects described to date [16,31].

The measures taken also translated into a financial effect. The economic analysis showed huge benefits in terms of the profitability of the ward’s work. A comparison of the parameter assessing the percentage of costs to the revenue generated emphatically confirmed the economic value of the introduced protocol. Despite the skyrocketing costs of patient treatment in recent years, the solutions applied translated into significant savings, and part of the funds obtained were used by hospital management to subsidise staff training and to modernise and expand the ward’s equipment resources. Reducing the length of hospitalisation, shortening the time a patient spends in the operating theatre, and the standardisation and optimisation of drug policy are all the tangible benefits in terms of improving the quality of patient treatment, but also the profitability and viability of ward operations. This is of paramount importance during a time of global problems such as the huge increase in patient treatment costs observed in recent years. We are of the opinion that today, in the fight for patients’ health, the economic aspects of new solutions in medicine should be emphasised more and more often, and should allow highly specialised hospitals, which today often face huge financial problems, to maintain their operations.

In the field of paediatric orthopaedics, there are few studies showing the financial translation of protocolised care [3,4,10]. As the economic analyses of the presented study showed, the reduction of hospitalisation time and the standardisation of the perioperative care process allowed a reduction in treatment costs. At the same time, an increase in the number of operations performed allowed an increase in income from contracted services. Reducing the length of patients’ hospital stays made it possible to increase the number of procedures performed at the clinic and reduce the waiting queue for elective orthopaedic procedures. The optimisation of perioperative care based on the principles of the ERAS protocol has also allowed more surgical procedures to be performed during a single day.

The application of ERAS recommendations in practice is sometimes difficult in every country mainly due to organisational reasons and insufficient staffing. Operating in haphazardly selected treatment teams, whose members change frequently, does not improve working comfort or patient safety. It was shown that the quality of work in permanent surgical teams, whose members have good relationships and know each other well, has a positive impact on the final outcome of the patient’s treatment [32,33]. As an example, the centre where this project was developed and carried out confirms these observations. Furthermore, it shows that adapting the ERAS guidelines and creating a local interdisciplinary management protocol in the setting of Polish paediatric orthopaedics is possible and can be implemented in other countries. In this way, tangible benefits can be achieved in the expected areas such as the reduction of complications, the reduction of hospitalisation time and costs, and increased satisfaction of patients, families, and medical staff.

Our study has some limitations. One of them is the known issues regarding observational pre/post-study design. Next is the parameter determining the frequency of rehospitalisation. It is not possible to compare the number of all-cause rehospitalisations in the face of performing more complex and complicated procedures. Although a higher frequency was observed since the introduction of the new surgical procedures, this did not have a negative impact on the unit’s financial results.

When the topic of ERAS is described, the RECOvER checklist is useful [34]. However, some points of the checklist are not fulfilled or possible to report on our study, e.g., we cannot define primary or secondary outcomes, or inclusion or exclusion criteria because of the before–after study design. Once we created the BOF app, we included it in our routine practice without waiting for the modification of the electronic survey or validation of this tool. Some detailed data about compliance were not reported. However, our study was designed to assess how to match some points of established ERAS protocol to polish conditions (e.g., education and prehabilitation).

## 5. Conclusions

The optimisation of perioperative management in paediatric orthopaedics based on novel tools and the interdisciplinary ERAS protocol is possible and brings tangible benefits in psychological, organisational, and financial terms and, above all, has a positive impact on the final outcome of treatment.

## Figures and Tables

**Table 1:** Interventions and tools adopting the ERAS protocol.

ERAS	Our Project
PATIENT before surgery	
Preoperative preparation and consultation No prolonged starvation Preoperative supply of carbohydrate-rich drink No premedication	Preparation in the Outpatient Orthopaedic and Anaesthesiology Clinic BOF^®^ educational and training application (prehabilitation) Protocol on preoperative fasting in accordance with the guidelines [17] Pre-op (Nutricia, Poland) Minimal premedication and distractors (soap bubbles, cartoons)
Before surgery TEAM	
Regular training to use minimally invasive techniques	Regular training of the interdisciplinary team
ANAESTHETIST during surgery	
Prevention of thromboembolic complications Prophylactic antibiotic therapy Prevention of hypothermia Short-acting anaesthetics Multimodal analgesia Prevention of postoperative nausea and vomiting Fluid and electrolyte balance	Protocol covering antibiotic prophylaxis, thromboembolic complications, postoperative nausea, and vomiting [18] Analgesia in advance Compliance with the guidelines of multimodal analgesia [19] with particular emphasis on regional anaesthesia and cryoanalgesia Advanced haemodynamic monitoring for extensive treatments to optimise among others normovolemia and normothermia [20]
ORTHOPAEDIC during surgery	
Training in minimally invasive techniques	Personalised operation plan Focus on minimally invasive techniques
After surgery TEAM	
Avoidance or short-term maintenance of catheters and drains Continuation of multimodal analgesia Early mobilisation and rehabilitation Early return to oral nutrition	These recommendations are included in our protocol

**Table 2:** Comparison of parameters analysed.

	BEFORE Group (*n* = 1553)	AFTER Group (*n* = 2545)	*p*
Age groups n (%)			
<1 year old	23 (2)	22 (1)	<0.001
1–6	193 (12)	223 (9)	
7–18	1337 (86)	2300 (90)	
Gender n (%)			
Girls	610 (39)	1052 (41)	0.19
Boys	943 (61)	1493 (59)	
Category n (%)			
Category 1	261 (17)	881 (34)	0.005
Category 2	44 (3)	198 (8)	
Category 3	7 (<1)	73 (3)	
Category 4	1241 (79)	1393 (55)	
Duration of hospitalisation (days, mean ± SD)			
Whole group (n = 4098)	4.76 ± 3.92	1.88 ± 2.22	<0.001
Category 1 (n = 1142)	6.09 ± 3.60	1.81 ± 0.73	<0.001
Category 2 (n = 242)	6.88 ± 2.30	2.25 ± 1.09	
Category 3 (n = 80)	16.86 ± 7.14	7.14 ± 9.84	

Legend: n—number of patients, SD—standard deviation.

**Table 3:** Percentage of costs to revenue in years.

Year	2018	2019	2020	2021	2022
Cost/revenue	85.02%	81.09%	84.83%	67.97%	71.15%

## Data Availability

The raw data are available upon request from the corresponding author.
